# Robotic repair of perineal hernias: a video vignette and review of the literature

**DOI:** 10.1007/s00464-022-09521-2

**Published:** 2022-08-18

**Authors:** Sarah Watanaskul, Marisa E. Schwab, Alexis Colley, Hueylan Chern, Madhulika G. Varma, William Y. Hoffman, Ankit Sarin

**Affiliations:** 1 School of Medicine, University of California San Francisco, San Francisco, CA USA; 2 Department of Surgery, University of California San Francisco, 550 16th Street, San Francisco, CA 94143 USA; 3 Department of Plastic Surgery, University of California San Francisco, San Francisco, CA USA

**Keywords:** Robotic, Perineal hernia, MIS, Da Vinci

## Abstract

**Background:**

Perineal hernias can be secondarily acquired following abdominoperineal resection of the rectum. While transabdominal minimally invasive techniques have traditionally used laparoscopy, there are few studies published on the robotic platform, which has been gaining popularity for other types of hernia repairs. We review the existing literature, share a video vignette, and provide practical tips for surgeons interested in adopting this approach.

**Methods:**

A literature search in Pubmed was performed to include all articles in English describing robotic repair of perineal hernias with identification of variables of interest related to repair. A case presentation with an accompanying video vignette and lessons learned from the experience are provided.

**Results:**

Seven case reports (four containing video) published between 2019 and 2022 were included. Most articles (*n* = 5) utilized the Da Vinci Si or Xi, and most patients (*n* = 5) had undergone abdominoperineal resection with neoadjuvant chemotherapy to treat rectal cancer. Patients were positioned in Trendelenburg with rightward tilt (*n* = 2), modified lithotomy (*n* = 1), or a combination of the two (*n* = 1). All articles (*n* = 7) reported closing the defect and using mesh. Three articles describe placing five ports (one camera, three robotic, one assistant). There were no significant intraoperative or postoperative complications reported, and no recurrence noted at 3–27 months follow-up. Based on our experience, as shown in the video vignette, we recommend lithotomy positioning, using porous polypropylene mesh anchored to the periosteum of the sacrum and peritoneum overlying the bladder and side wall, and placing a drain above the mesh.

**Conclusions:**

A robotic transabdominal approach to perineal hernia repair is a viable alternate to laparoscopy based on low complication rates and lack of recurrence. Prospective and longer duration data are needed to compare the techniques.

**Supplementary Information:**

The online version contains supplementary material available at 10.1007/s00464-022-09521-2.

Perineal hernias are formed when there is a defect in the pelvic floor, which allows for the protrusion of intra-abdominal contents through the perineum [1]. Secondary perineal hernias occur after pelvic surgery and most commonly occur following abdominoperineal resection of the rectum (APR). Historically, the cited complication rate for standard APR was 1% [2]; however, with the advent of extralevator APR the rates of perineal hernia have been described as high as 26% [3]. Perineal hernias can be surgically repaired via a perineal or abdominal approach or a combined abdominal and perineal approach [4].

The robotic platform has been increasingly used for various types of abdominopelvic surgery. Perineal hernia repair is well suited for the robotic approach given the excellent 3-D visualization, ability to work in a limited space and precise suturing of mesh to close defects. However, only a handful of institutions have published their experience using the robotic platform [5–11]. In this study, we sought to review the existing literature on robotic repair of perineal hernias, provide a video vignette that illustrates the principles of robotic repair, and provide practical tips for surgeons interested in adopting a robotic approach.

## Materials and methods

A literature search was performed using a combination of the following search terms in Pubmed: “perineal hernia”, “robot”, “repair.” Inclusion criteria were primary articles in English describing robotic repair of perineal hernias. Exclusion criteria were review articles, articles describing other robotic pelvic surgeries, and articles describing non-robotic approaches to perineal hernia repair. The included articles were analyzed, and the following variables were tabulated for the purpose of the review: sample size, study design, robotic platform, indication, patient positioning, port placement, mesh, and complications.

A case presentation of a patient with a perineal hernia was described, along with a video vignette. The operative details of the case and tips gained from this experience were provided.

This study was approved by the Institutional Review Board (IRB) at the University of California, San Francisco (UCSF) Study Number 18-26677.

## Results

### Literature review

Seven articles published between 2019 and 2022 were included (Table 1). All seven of the included articles were single patient case reports, and four articles contained an accompanying video. Robot types included the Da Vinci Si (*n* = 3) and Da Vinci Xi (*n* = 2); two articles did not specify which version of the Da Vinci system they used.

**Table 1:** Literature review

Author	Sample size	Study design	Robotic Platform	Indication	Positioning	Port Placement	Mesh	Complications
Rajabaleyan [5]	1	Case report	Da Vinci Si	Hx laparoscopic intersphincteriec proctocolectomy with permanent ileostomy, with perineal bulge	Trendelenburg, tilted right	Camera above and to right of umbilicus. Three ports (RLQ, LLQ, LUQ) and assisting port between the camera and RLQ	Symbotex Composite coated monofilament polyester	None
Maurissen [6]	1	Case report	Da Vinci Xi	Hx laparoscopic extralevator APR, with sharp perineal pain and swelling	Modified lithotomy	3 8 mm trocars at umbilical level towards R side. 5 mm assistant port	Non-absorbable synthetic Symbotex	Asymptomatic seroma, treated conservatively
Avondstondt [7]	1	Case report, video vignette	Not stated	Hx 4 vaginal deliveries, with posterior perineal hernia and stage III uterovaginal prolapse	–	–	Polypropylene type 1 “Y” mesh	None
Li [8]	1	Case report	Da Vinci Si	Hx APR with perineal bulge	Trendelenburg, tilted right	Camera 2 cm above umbilicus. Three ports (RLQ, LLQ, LUQ) and assisting port in RUQ	Non-absorbable synthetic	None
Pramateftakis [9]	1	Case report, video vignette	Da Vinci Si	Hx laparoscopic APR, with recurrent perineal hernia	–	–	Phasix ST	None
Glanzer [10]	1	Case report	Da Vinci	Hx 2 vaginal deliveries and vaginal hysterectomy, with rectal hernia through pelvic floor (fecal urgency, incontinence)	–	Trocars in transverse fashion at level of umbilicus	Synthetic bioabsorbable Phasix	None
Genovese [11]	1	Video correspondence	Da Vinci Xi	Hx robotic APR, symptomatic perineal hernia	Lithotomy and Trendelenburg	4 robotic trocars, 1 assistant trocar	Partially absorbable coated Proceed Mesh	None

*APR* abdominoperineal resection; *Hx* history; *LUQ* left upper quadrant; *RLQ* right lower quadrant; *RUQ* right upper quadrant; – indicates the variable was not included in the article

The majority of case reports involved patients who had undergone an APR (*n* = 5). Half of the studies commented on patient positioning, opting for Trendelenburg with rightward tilt (*n* = 2), modified lithotomy (*n* = 1) or a combination of the two (*n* = 1). The remaining three studies did not specify patient positioning. All authors successfully closed the defect and none required conversion to laparoscopy or open.

None of the studies reported significant perioperative or postoperative complications, and no recurrence was noted in any of the case reports following robotic perineal hernia repair, with follow-up appointment times ranging from 3 to 27 months. One study reported a postoperative asymptomatic seroma that was treated conservatively.

### Video vignette

**Fig. 1:**
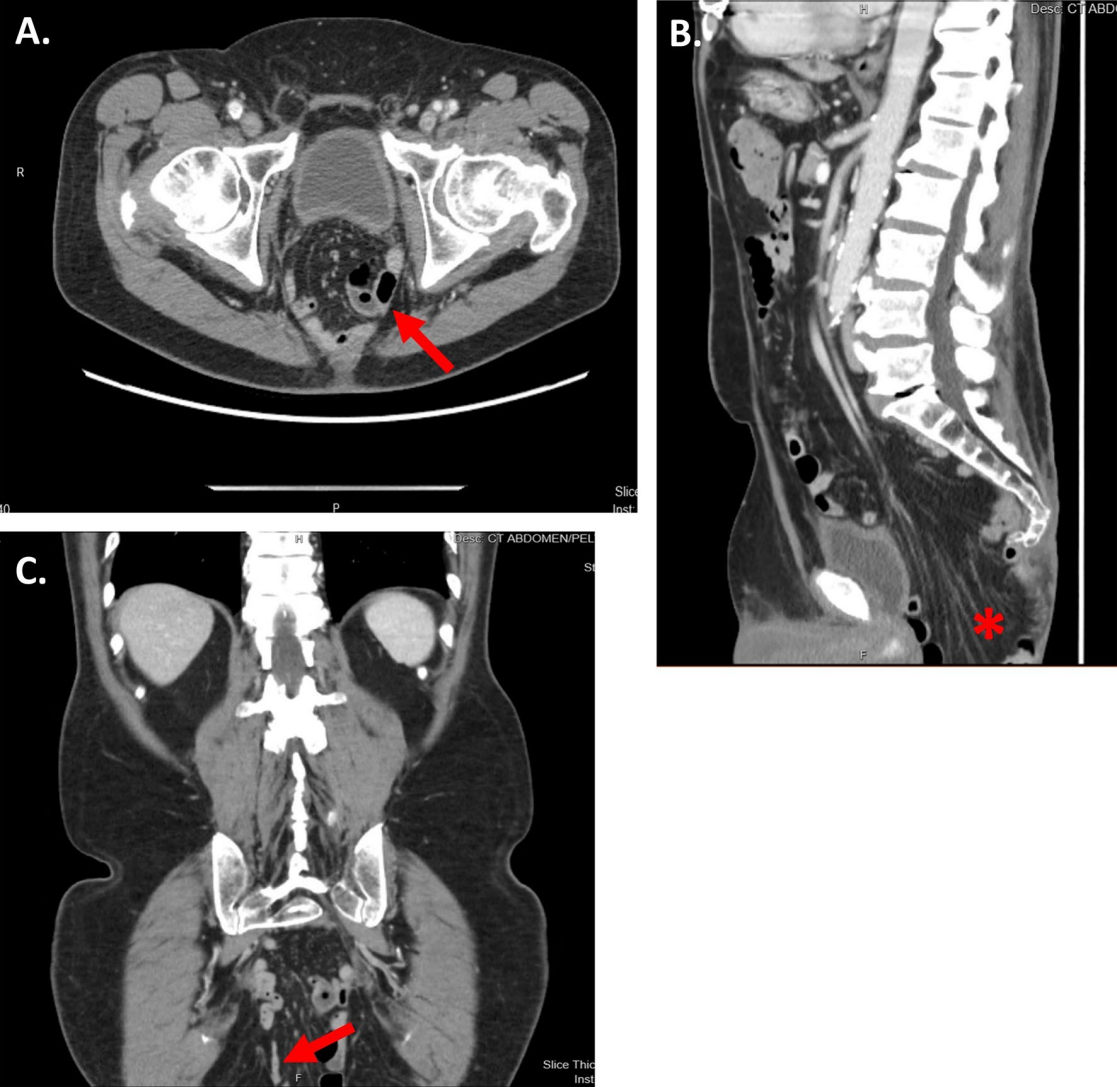
Pre-operative CT abdomen/pelvis showing the hernia defect (red arrow and star) in the **A** Axial view, **B** Sagittal view, and **C** Coronal view (Color figure online)

**Fig. 2:**
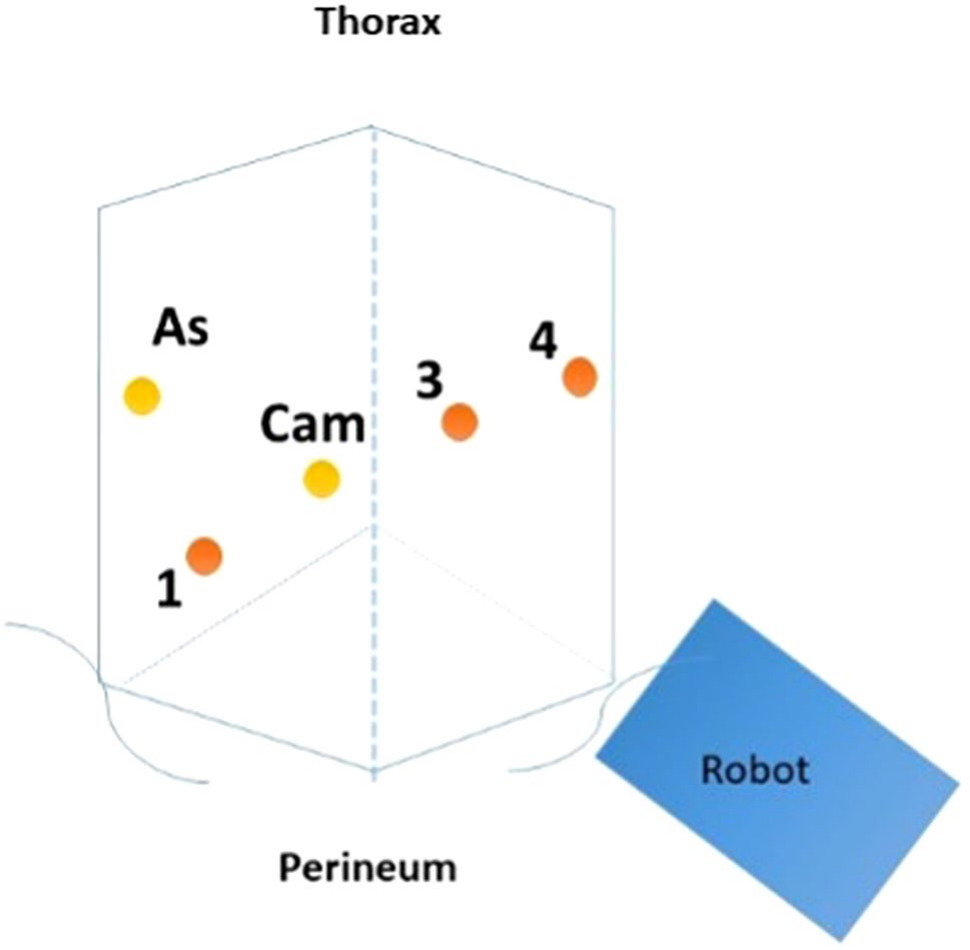
Diagram of the placement of the robotic trocars: four 8 mm robotic trocars are placed in a straight line from the RLQ to the LUQ spaced 7 cm apart including the Camera (Cam), with an AirSeal trocar (As) in the RUQ

The patient is a 70-year-old man with a history of rectal cancer invading the prostate who underwent a laparoscopic sigmoid colostomy with mucous fistula and neoadjuvant systemic chemotherapy and radiation. This was followed by a bladder-preserving robotic abdominoperineal resection and prostatectomy with gracilis muscle flap placement. Two years later, he presented with prolapsing tissue in the perineum that caused discomfort while sitting, and he was diagnosed with a perineal hernia. Preoperative CT scans showed a moderate sized perineal hernia containing small bowel on axial, coronal, and sagittal cross-sections (Fig. 1), and he was scheduled for a robotic dissection of the pelvis with reduction of small bowel followed by perineal hernia repair. A transabdominal approach was selected to preserve the gracilis muscle flap placed during the prior operation. The steps of the procedure are outlined in the accompanying video (see linked video). The patient was placed in a low lithotomy position with Allen stirrups. Four 8 mm robotic trocars were placed in a straight line from the right lower quadrant (RLQ) to the left upper quadrant (LUQ) spaced 7 cm apart and an assistant 5 mm AirSeal trocar in the right upper quadrant (RUQ) (Fig. 2). Arm 1 of the robot in the RLQ had scissors, arm 2 adjacent to the umbilicus had the camera, and arms 3 and 4 in the two left-sided ports had the fenestrated and tip-up grasper. The estimated blood loss was 50 mL and the Foley was removed at the end of the case. There were no intraoperative complications, and the patient was discharged home on postoperative day 2. At 2 and 6 months follow-up, the patient was doing well and no recurrence was noted on physical exam.

## Discussion

Given the relative rarity of perineal hernias, it is not surprising that the literature on robotic perineal hernia repair consists of only case reports. Despite the small sample size, the lack of reported complications, need for conversion or hernia recurrence albeit in limited follow up make the robotic platform a promising modality to repair perineal hernias, particularly those that occur secondary to abdominoperineal resection of the rectum.

Of note, the variables reported in the case reports are not standardized; for example, some studies commented on patient positioning, operative time, and/or port placement whereas others did not. Future studies on the efficacy of a robotic approach to perineal hernia repair may benefit from pooling data across institutions to increase the effective sample size, as well as establishing a consistent set of reported variables.

In our accompanying video vignette, we present our approach to this technique. We opted to position our patient in lithotomy in case there was a need for a perineal approach in addition to the robotic abdominal approach. Dissection and reduction of the hernia is straightforward using robotic technique. We used a porous, polypropylene mesh to close the defect and recommend placing the drain above the mesh. In doing so, it allows the drain to absorb fluid through the mesh while keeping the perineum isolated from the abdomen. One of the critical aspects of the case is identifying the appropriate tissue to anchor the mesh in the perineum. We recommend anchoring the mesh to the periosteum of the sacrum for strength, anteriorly to the peritoneum overlying the bladder, and laterally to the parietal peritoneum that covers the structures at the pelvic brim comprising the ‘side wall’—these include branches of the iliac vessels, splanchnic nerves and ureters. There is no ideal location to anchor sutures laterally in the pelvis. We anchor robustly to the periosteum posteriorly and a little less robustly by incorporating part of bladder muscle wall while anchoring to the peritoneum anteriorly. Laterally, we are limited to anchoring to the peritoneum alone in order to prevent direct or traction injury to the vessels, nerves and ureters in this location. The purpose of these lateral sutures is more to keep the mesh flat than for strength—the hope is that because the repair is high in the pelvis, even if the peritoneum will get stretched due to the weight of the bowel this will be limited to the deep pelvis and not present as a perineal hernia.

Since almost universally, perineal hernias occur after abdominoperineal resection of the rectum (with the rectum absent), the visualization is typically adequate for both ureters at the pelvic brim. If there is a concern for visualization, use of ureteral stents with indocyanine green and immunofluorescence [12] would be reasonable, although we did not feel it was needed for this case. We are also careful to only take peritoneum when suturing laterally to avoid injury.

In the limited literature available, the recurrences with this approach favor comparably to other (perineal) approaches. We need longer term data to understand if this strategy is adequate. A systematic review of perineal hernia repairs after abdominoperineal excision or extralevator abdominoperineal excision revealed a primary recurrence rate of 24.1% (26/108) and second recurrence rate of 26.9% (7/26) [13]. Further studies are needed to understand the recurrence rate after abdominal versus perineal repair of perineal hernias.

In conclusion, the robotic approach to a perineal hernia repair appears to be a reasonably effective alternative to laparoscopy and may have advantages over other approaches. Further investigation and collation of data is required to establish a difference compared to other approaches.

## Supplementary Information

Below is the link to the electronic supplementary material.

Supplementary file1 (MP4 177607 kb)

## References

[1] Moshcowitz AV (1916). Perineal hernia. Surg Gynecol Obstet.

[2] McKenna NP, Habermann EB, Larson DW (2020). A 25 year experience of perineal hernia repair. Hernia.

[3] Sayers AE, Patel RK, Hunter IA (2015). Perineal hernia formation following extralevator abdominoperineal excision. Colorectal Dis.

[4] Hung LY, Abbass MA, Sapci I (2021). Surgical repair of postoperative perineal hernia: a case for the perineal approach. Dis Colon Rectum.

[5] Rajabaleyan P, Dorfelt A, Poornoroozy P (2019). Robot-assisted laparoscopic repair of perineal hernia after abdominoperineal resection: a case report and review of the literature. Int J Surg Case Rep.

[6] Maurissen J, Schoneveld M, Van Eetvelde E (2019). Robotic-assisted repair of perineal hernia after extralevator abdominoperineal resection. Tech Coloproctol.

[7] Avondstondt AM, Ezzedine D, Salamon C (2019). Perineal hernia repair using permanent suture and mesh: a video case presentation. Int Urogynecol J.

[8] Li D, Zhang S, Zhang Z (2020). A new method of robot-assisted laparoscopic repair of perineal hernia after abdominoperineal resection: a case report. Int J Colorectal Dis.

[9] Pramateftakis MG, Kotidis E, Gkantsinikoudis N (2020). Robotic-assisted repair of perineal hernia after laparoscopic abdominoperineal excision using a bioresorbable mesh—a video vignette. Colorectal Dis.

[10] Glanzer R, O’Neil B, Turaihi H (2021). Pararectal hernia: literature review and surgical repair techniques in the era of robotic surgery. J Surg Case Rep.

[11] Genovese A, Giuliani G, Formisano G (2022). Robotic perineal hernia repair with lateral mesh suspension—a video vignette. Colorectal Dis.

[12] Soriano CR, Cheng RR, Corman JM (2022). Feasibility of injected indocyanine green for ureteral identification during robotic left-sided colorectal resections. Am J Surg.

[13] Balla A, Batista Rodriguez G, Buonomo N (2017). Perineal hernia repair after abdominoperineal excision or extralevator abdominoperineal excision: a systematic review of the literature. Tech Coloproctol.

